# Pediatric Secondary Transfer Percentages: A Retrospective Observational Study

**DOI:** 10.7759/cureus.6766

**Published:** 2020-01-24

**Authors:** Yves Leroux, Jolene Cook, Judah Goldstein, Steve Doucette, Corinne DeMone, Alix Carter, Katrina F Hurley

**Affiliations:** 1 Emergency Medicine, Dalhousie University, Halifax, CAN; 2 Research Methods Unit, Nova Scotia Health Authority, Halifax, CAN; 3 Emergency Medicine, Nova Scotia Health Authority, Halifax, CAN; 4 Division of Emergency Medical Services, Dalhousie University, Halifax, CAN; 5 Emergency Department, IWK Health Centre, Halifax, CAN

**Keywords:** pediatrics, transportation of patients, patient transfer, emergency medical services

## Abstract

Introduction: Certain adult conditions treated by paramedics, such as myocardial infarction or stroke, have better outcomes if transported to a specialty centre, bypassing local generalist facilities when necessary. Little evidence exists to inform guidelines to identify pediatric patients who would benefit from direct transport to a pediatric centre. This study describes the characteristics of children brought to community emergency departments (ED) who subsequently required transfer to pediatric specialty care.

Methods: A retrospective observational cohort study was performed in a metropolitan area with one tertiary pediatric specialty centre and four community EDs. The patient care record database was queried for patients under 16 years old transported by paramedics to a community ED during a five-year period. Secondary transfer to the pediatric specialty centre within 24 hours was identified. The primary outcome was percentage of transfers to specialty care. Descriptive statistics were used to characterize the whole group as well as stratified by age category, chief complaint and Canadian Triage Acuity Scale (CTAS).

Results: A total of 872 pediatric patients were transported to community EDs with 95 (10.9%) requiring secondary transfer to the pediatric specialty centre. CTAS 1 and 2 were associated with increased secondary transfer (p<0.001). There were also differences in transfer proportion by chief complaint. There was no association between age or gender and transfer to pediatric specialty care.

Conclusions: This retrospective study shows an association between acuity and certain chief complaints and percentage of secondary transfer to pediatric specialty care.

## Introduction

Regionalized systems of healthcare are used to improve the outcomes of patient populations requiring specialized treatment. The integration of prehospital care providers relies on clinical guidelines that allow these patients to be identified appropriately in the field and transported directly to the required resource, bypassing other destinations. In adults, guidelines that enabled paramedics to identify patients with ST-elevation myocardial infarction, notify the cardiology team and transport the patient directly to the regional specialty centre led to a decreased mortality from 8.9% to 1.9% compared to transporting to the nearest hospital [1].

Specialized pediatric centres currently exist throughout North America. Other than traumatic injury, there is a paucity of evidence to inform clinical guidelines that would assist prehospital identification of those patients who would benefit most from direct transport to the pediatric centre instead of the nearest hospital. This knowledge gap has recently been identified as a priority study objective for pediatric prehospital research [2]. Children brought by paramedics to a community emergency department (ED) and subsequently transferred to a tertiary care pediatric hospital may benefit from direct transport from the scene to a pediatric specialty centre.

Study objective

Our objective was to describe the clinical characteristics of children brought to community EDs who subsequently required transfer to pediatric specialty care.

## Materials and methods

Study design and setting

This retrospective observational study was performed in a metropolitan area served by one tertiary care pediatric hospital which sees patients aged 0-15 years old and four community EDs serving all age groups. Prehospital care and transport is performed by a single Advanced Life Support province-wide Emergency Medical Services (EMS) system. This sample consisted of all patients less than 16 years of age transported by paramedics to any of the four community EDs between January 1, 2010 and December 31, 2015.

Data search

The EMS system maintains a database of dispatch records and electronic patient care records for every request for ambulance service in the province. The search process identified all patients who met the eligibility criteria. Deterministic linkage using first name, last name, sex, date of birth, health card number, date of service and facility address was used to search for subsequent interfacility transfer within 24 hours.

Outcome measures

The primary outcome was the proportion of patients requiring subsequent transfer to the pediatric specialty centre within 24 hours of being transported from the field to a community hospital by paramedics (secondary transfer). Secondary outcomes were the proportion of transfers by age, chief complaint and Canadian Triage and Acuity Scale (CTAS).

Data analysis

Baseline demographics were summarized as means and standard deviation for continuous data. Categorical data were summarized as proportions with percentages. Chief complaints were grouped into categories. Secondary transfer proportions in each chief complaint category were shown as percentages. The effect of age, gender, and CTAS category on transfer to the specialty centre were shown as odds ratios (OR) with 95% confidence intervals (CI). P-values less than 0.05 were considered statistically significant. All analyses were performed using SAS, version 9.4 (SAS Institute, Cary, NC).

Ethics

This study was approved by the Nova Scotia Health Authority Research Ethics Board (File No. 1021022).

## Results

During the five-year study period, 872 pediatric patients (mean age 9.7 years ± 4.90) were transported by paramedics to a community ED. Of these, 95 (10.9%) patients were subsequently transferred to the pediatric specialty centre (Table 1).

**Table 1:** Demographic data of the study population CI, confidence interval; CTAS, Canadian Triage Acuity Scale

Characteristic	No Secondary Transfer (n=777, 89.1%)	Secondary Transfers (n=95; 10.9%)	All Patients (n=872)
Age, mean (95% CI)	9.7 years (0.1-19.3)	9.8 years (1.1-18.4)	9.7 years (0.1-19.3)
Male (n, %)	406 (52.2%)	55 (57.9%)	461 (52.8%)
CTAS (n, %)			
CTAS 1	2 (0.3%)	7 (7.4%)	9 (1.0%)
CTAS 2	92 (11.8%)	33 (34.7%)	125 (14.3%)
CTAS 3	377 (48.5%)	38 (40.0%)	415 (47.6%)
CTAS 4	231 (29.7%)	14 (14.7%)	245 (28.1%)
CTAS 5	65 (8.4%)	2 (2.1%)	67 (7.7%)

A total of 461 (52.8%) patients were male with no significant difference in odds of transport (p=0.48). Nearly half of all patients were triaged as CTAS 3 (n=415; 47.6%). There was a clear trend in increasing odds of transfer as CTAS decreased, with a statistically significant odds ratio for CTAS 2 (OR: 11.6, 95% CI: 2.68-50.3, p=0.001) and CTAS 1 (OR: 113.4, 95% CI: 13.6-941.2, p<0.0001) relative to CTAS 5 (Table 2).

**Table 2:** ORs of transfer by CTAS, sex and years of age OR, odds ratio; CI, confidence interval; CTAS, Canadian Triage Acuity Scale

	OR (95% CI)	P-Value
CTAS		
1	113.36 (13.64, 941.8)	<0.0001
2	11.61 (2.68, 50.33)	0.001
3	3.28 (0.77, 13.94)	0.11
4	1.97 (0.44, 8.92)	0.38
5	Reference	Reference
Male vs. female	1.18 (0.75, 1.86)	0.48
Age (per year)	1 (0.96, 1.05)	0.9

There was no significant trend in transfer by age (p=0.9). There were only four patients younger than one year brought to a community ED, none of whom were subsequently transferred.

The 36 chief complaints in the sample were grouped into 16 categories for analysis. The most common complaint was “minor trauma” (n=406). Categories of “major trauma”, “diabetic complaint”, “altered mental status” and “overdose/poisoning” had the highest percentage of secondary transfer at 50%, 50%, 41.7% and 24.4%, respectively (Table 3).

**Table 3:** Proportion of secondary transfers by chief complaint

Chief Complaint	Overall	Transferred (n=95)	Not Transferred (n=777)	Proportion Transferred	P-Value
Pain/nausea	67 (7.7%)	<5 (<5%)	63 (8.1%)	5.97%	
Allergic reaction	27 (3.1%)	<5 (1.1%)	26 (3.4%)	3.70%	
Cardiovascular	31 (3.6%)	<5 (1.1%)	30 (3.9%)	3.23%	
Altered mental status	12 (1.4%)	5 (5.3%)	7 (0.9%)	41.67%	
Minor trauma	406 (46.6%)	39 (41.1%)	367 (47.2%)	9.61%	
Major trauma	8 (0.9%)	4 (4.2%)	4 (0.5%)	50.00%	
Environmental	2 (0.2%)	0 (0%)	2 (0.3%)	0.00%	
Obstetrics	1 (0.1%)	0 (0%)	1 (0.1%)	0.00%	
Diabetic problem	2 (0.2%)	1 (1.1%)	1 (0.1%)	50.00%	
Wellness check	74 (8.5%)	3 (3.2%)	71 (9.1%)	4.05%	
Other	44 (5.1%)	4 (4.2%)	40 (5.2%)	9.09%	
Respiratory	44 (5.1%)	5 (5.3%)	39 (5%)	11.36%	
Overdose/poisoning	41 (4.7%)	10 (10.5%)	31 (4%)	24.39%	
Psychological	27 (3.1%)	4 (4.2%)	23 (3%)	14.81%	
Neuro	78 (8.9%)	14 (14.7%)	64 (8.2%)	17.95%	
Transfer	8 (0.9%)	0 (0%)	8 (1%)	0.00%	

## Discussion

This retrospective, observational cohort study found that increasing acuity and certain chief complaints had more frequent secondary transfer to pediatric specialty care within 24 hours.

“Major trauma” had a 50.0% proportion of secondary transfer, which was tied for highest. Decreased mortality in children brought directly to pediatric trauma centres has been demonstrated previously, and many EMS systems, including the one serving the study area, have destination guidelines for pediatric trauma [3]. Although additional education about pediatric field triage guidelines may help providers to appropriately transport major trauma patients directly to the pediatric trauma centre, the paramedics who transported these trauma patients may have had justification for deviating from destination policy, such as requiring assistance with an airway.

“Diabetic complaint” was found to have a secondary transfer proportion of 50.0% although there were only two patients with this chief complaint transported to community hospitals during the study period. It is unclear if the incidence of pediatric diabetic issues is low or if these patients were being informally directed towards the pediatric centre. Knowlton et al. previously demonstrated that chronic medical conditions, such as diabetes, are linked to increased ambulance usage, but there are no published studies examining whether these patients benefit from specialized care [4].

“Altered mental status” had the third-highest transfer proportion (41.7%). A variety of infectious, neurologic, metabolic or toxicologic causes can lead to this presentation which entails significant cerebral dysfunction. Prehospital level of consciousness may be a meaningful although non-specific indicator of severity in pediatric medical illness.

Patients transported to community hospitals with a complaint of “overdose/poisoning” had a transfer proportion of 24.4%. Epidemiologic studies have found that the majority of these presentations are intentional ingestions and intoxication [5,6]. These patients may require mental health assessment which is only available at the pediatric specialty centre in our community. A recent study of adolescents visiting EDs in the United States found a sixfold increase in the odds of transfer or admission for those presenting with mental health complaints. Further study may be of benefit since pediatric patients with mental health and substance abuse have a higher proportion of repeat EMS usage [4].

## Conclusions

In our retrospective study, pediatric patients transported by EMS to a community hospital with complaints of major trauma, diabetic complaint, altered mental status or overdose/poisoning were subsequently transferred to tertiary pediatric care more frequently than other chief complaints. Higher acuity patients, based on the CTAS score, also had a significantly higher percentage of secondary transfer to specialized pediatric care. These findings may help derive a decision tool to identify which pediatric patients would benefit from bypassing closer EDs in favour of transport directly to pediatric specialty care.
